# Hepatokine Profile in Adolescents with Polycystic Ovary Syndrome: A Case–Control Study

**DOI:** 10.3390/jcm12175744

**Published:** 2023-09-04

**Authors:** Aikaterini Giannouli, Charikleia Stefanaki, Christos Kouskoutis, Marianna Konidari, Iliana Mani, Konstantina Konidari, Sophia L. Markantonis, Aimilia Mantzou, Spyridon P. Dourakis, Efthymios Deligeoroglou, Flora Bacopoulou

**Affiliations:** 1 Center for Adolescent Medicine and UNESCO Chair in Adolescent Health Care, First Department of Pediatrics, Medical School, National and Kapodistrian University of Athens, Aghia Sophia Children’s Hospital, 11527 Athens, Greece; giannouli.katerina@gmail.com (A.G.); cstefanaki@gmail.com (C.S.); konna.kon13@gmail.com (K.K.); 2 Laboratory of Biopharmaceutics and Pharmacokinetics, Department of Pharmaceutical Technology, Faculty of Pharmacy, National and Kapodistrian University of Athens, 15771 Athens, Greece; ckouskoutis@pharm.uoa.gr (C.K.); kyroudi@pharm.uoa.gr (S.L.M.); 3 Department of Radiology, Medical School, National and Kapodistrian University of Athens, Aretaieio Hospital, 11528 Athens, Greece; marianna_konidari@hotmail.com; 4 Second Department of Internal Medicine, Medical School, National and Kapodistrian University of Athens, Hippokratio Hospital, 11527 Athens, Greece; ilianamani@windowslive.com (I.M.); spdour@med.uoa.gr (S.P.D.); 5 Division of Endocrinology, Metabolism, and Diabetes, First Department of Pediatrics, School of Medicine, National and Kapodistrian University of Athens, Aghia Sophia Children’s Hospital, 11527 Athens, Greece; amantzou@med.uoa.gr; 6 Department of Pediatric & Adolescent Gynecology, Mitera Children’s Hospital, 15123 Athens, Greece; deligeoroglou@yahoo.gr

**Keywords:** polycystic ovary syndrome, PCOS, NAFLD, liver, hepatokines, fatty liver disease, SHBG, selenoprotein, FGF21, fetuin, adolescents, Greece

## Abstract

The current guidelines suggest routine screening for non-alcoholic fatty liver disease (NAFLD) in patients with polycystic ovary syndrome (PCOS). Hepatokines seem to be promising surrogate endpoints for the diagnosis and severity of NAFLD. PCOS has its onset in adolescence and its metabolic sequalae begin during the same period. There are scarce data on the hepatokine profile of adolescent PCOS patients. This case–control study examined the serum profile of the hepatokines sex hormone-binding globulin (SHBG), selenoprotein P, fibroblast growth factor 21 (FGF21), and fetuin A in a sample of adolescent PCOS patients, and their association to metabolic and hormonal parameters. The selenoprotein P and SHBG serum concentrations were significantly decreased in PCOS patients vs. the controls (median (IQR), 2.47 (0.40) vs. 2.66 (0.36) μg/mL, *p* = 0.025; mean ± SD, 41.71 ± 19.41 vs. 54.94 ± 22.12 nmol/L, *p* = 0.011, respectively), whereas selenoprotein P was significantly and positively associated with testosterone (r = 0.325, *p* = 0.007) and the free androgen index (r = 0.361, *p* = 0.002). The SHBG demonstrated multiple significant negative correlations with adverse metabolic parameters. Among the PCOS patients, the FGF21 concentrations were significantly higher in those with NAFLD, whereas a 1 pg/mL increase in the FGF21 concentration increased the odds of NAFLD diagnosis by liver ultrasound by 1%, suggesting FGF21 as a potential biomarker for hepatic disease in females with PCOS in adolescence. Fetuin A was the least differentiated hepatokine between the PCOS patients and controls with the least associations with metabolic and hormonal parameters.

## 1. Introduction

Polycystic ovary syndrome (PCOS) is a constellation of symptoms and signs with different phenotypes, affecting 6% to 20% of patients of reproductive age, depending on the geographic region. It is characterized by hyperandrogenism (HA), chronic anovulation, and polycystic ovarian morphology. PCOS patients seem to present with metabolic sequelae, often commencing in adolescence [1]. Non-alcoholic fatty liver disease (NAFLD) has been strongly associated with PCOS, with a three-fold prevalence in PCOS patients, when compared with healthy controls [2,3]. In 2014, a routine assessment for NAFLD was incorporated in the standard follow-up of patients with PCOS, as a recommendation by the American Association of Clinical Endocrinologists (AACE) and the Androgen Excess and PCOS Society [4]. Several studies examining NAFLD in adolescents with PCOS have reported a wide range of prevalence (6.7–50%) depending on the special characteristics of each sample and the method used for the diagnosis of NAFLD [5,6,7,8,9,10,11].

Hepatokines are recently discovered organokines secreted by hepatocytes that act in the autocrine, paracrine, and endocrine systems. Various tissues use organokines to exert a vast number of metabolic actions. The liver secretes hepatokines, when needed, for metabolism and the availability of nutrients and energy to the central nervous system (CNS), the adipose tissue, and the muscles. The secretion of hepatokines is altered in patients with NAFLD, resulting in metabolic dysregulation [12]. During the last decade, many studies have demonstrated the implication of hepatokines in the pathophysiology of metabolic dysfunction and the onset of NAFLD. 

Sex hormone-binding globulin (SHBG) is a hepatokine mainly secreted by the liver that regulates the bioavailability of sex hormones [13,14]. SHBG has emerged as a metabolic biomarker; low SHBG levels were reported to be predictive for type 2 diabetes mellitus (T2DM) in males and females, and an inflammatory mediator in the pathogenesis of metabolic disease [14,15,16]. Biochemically, it is a transport carrier, binding estrogen and androgens and regulating their biological activity, and it is usually used as a surrogate endpoint for hyperandrogenism in adult patients with PCOS. Low serum SHBG concentrations are considered to be a biomarker of metabolic abnormalities and they are linked to insulin resistance (IR), hyperandrogenemia, hyperglycemia, and dyslipidemia in PCOS patients. SHBG is also related to the long-term prognosis of PCOS, whereas SHBG gene polymorphism is associated with PCOS risk [17]. 

Selenoprotein P (SeP), a hepatokine and a selenocysteine carrier [18,19,20], was first discovered in 1973 [21] and has known protective effects on lipid peroxidation. Other studies have revealed increased concentrations of SeP in patients with IR, T2DM, and NAFLD [16,22]. A causal relationship has just recently been established, both in vivo and in vitro, between SeP and IR in hepatocytes and myocytes [23]. 

Another hepatokine, the fibroblast growth factor 21 (FGF21), has been linked to NAFLD. FGF21 lacks a heparin binding site and seems to act as part of the endocrine system [24]. FGF21 has been associated with obesity, T2DM, and hepatic steatosis, even in pediatric populations [25]. Interestingly, FGF21 exogenous administration seems to improve obesity, insulin sensitivity, and reverse steatosis [12,26,27]. 

The hepatokine fetuin A acts as an adaptor between free fatty acids and toll-like receptor 4 (TLR4), activating proinflammatory pathways and contributing to IR [28]. Various researchers have found increased fetuin A concentrations in NAFLD animal models and in humans [29,30,31]. 

Conclusively, PCOS and NAFLD seem to be interconnected. Hepatokines seem to be reliable markers for the presence of NAFLD. Interestingly, to the best of our knowledge, no studies exist about the hepatokine profile in adolescent PCOS patients to date. The aim of this study was to examine the association of hepatokines with metabolic and hormonal parameters in adolescents with PCOS, along with the predictive ability of hepatokines for the presence of NAFLD in these patients. 

## 2. Materials and Methods

### 2.1. Study Design—Setting

This was a case–control study, conducted at the tertiary Center for Adolescent Medicine and UNESCO Chair in Adolescent Health Care, of the First Department of Pediatrics, School of Medicine, National and Kapodistrian University of Athens, at the Aghia Sophia Children’s Hospital in Athens, Greece, between January 2017 and December 2020. The study was conducted in agreement with the Helsinki Declaration for human studies and was approved by the Ethics Committee of the Aghia Sophia Children’s Hospital (protocol number 29661/23-12-16). Eligible participants were assessed on the basis of the inclusion and exclusion criteria and were informed about the procedures and the aim of the study. Written informed consent was obtained by the participants and their legal guardians.

### 2.2. Participants

PCOS patients were adolescents up to 21 years who sought medical assistance due to menstrual disorders and/or clinical hyperandrogenism, and for whom a PCOS diagnosis was established according to the Rotterdam criteria [32]. Furthermore, in consonance with the recommendations of the Pediatric Endocrine Societies (Table 1), only moderate to severe and persistent biochemical and/or clinical hyperandrogenism, menstrual irregularity (menstrual cycle of <21 days or >45 days) for 2 or more years after menarche, and increased ovarian volume instead of polycystic morphology on pelvic ultrasound were considered as the diagnostic criteria of PCOS in this study [33,34,35]. The controls were adolescents of the same age who presented for routine annual health care visits during the same period, at least two years after menarche, with normal menstruation and without hirsutism. The exclusion criteria included Wilson disease, α-1 antitrypsin deficiency, hepatitis, congenital portosystemic shunts, a history of drug ingestion (steroids, amiodarone, alcohol, methotrexate, ecstasy, l-asparaginase, vitamin E, valproate, tamoxifen, antiretrovirals), malnutrition, celiac disease, intestinal failure-associated liver disease, cystic fibrosis-associated liver disease, Mauriac syndrome, myopathic disorders, hyperprolactinemia, Schwachman syndrome, Cushing syndrome, adrenal or ovarian tumors, premature ovarian failure, hypothyroidism or hyperthyroidism, congenital adrenal hyperplasia, diabetes mellitus, lipodystrophies, or any inborn errors of metabolism. 

The groups were divided into subgroups for further analyses, i.e., lean vs. overweight/obese; with low vs. normal insulin sensitivity; and with vs. without NAFLD.

### 2.3. Variables—Procedures 

#### 2.3.1. Blood Parameters

Blood sampling was performed between the 3rd and 5th day of the menstrual cycle in menstruating adolescents or on a random day in amenorrhoeic ones, between 8:00 and 9:00 a.m. after overnight fasting. After clotting at room temperature and 10 min of centrifugation at 3000 g, the serum was isolated and analyzed on the same day for both the hormonal and biochemical parameters and stored at −80 °C pending enzyme-linked immunosorbent assay (ELISA) analysis. The biochemical and hormonal parameters were measured at the laboratory of the National and Kapodistrian University of Athens, at the Aretaieio Hospital. A chemiluminescent microparticle immunoassay (CMIA) was performed to determine the testosterone values and an electrochemiluminescent immunoassay (ECLIA) was utilized for SHBG. The SeP serum concentration was measured using a sandwich ELISA kit (Human Selenoprotein P1– Cloud-Clone Corp., Houston, TX, USA) with a minimum detectable concentration of 0.33 ng/mL, intra-assay CV < 10%, and inter-assay CV < 12%. Similarly, fetuin A was measured using a quantitative sandwich ELISA kit (Human Fetuin A—R&D Systems, Inc., Minneapolis, MN, USA) with a minimum detectable concentration of 0.16 ng/mL, intra-assay CV < 4.9%, and inter-assay CV < 8.4%. Lastly, a Human FGF-21 kit (Bio Vender, Brno, Czech Republic) with a minimum limit of detection 7 pg/mL and intra- and inter- assay CV < 3.5% was used to measure the FGF21 concentrations.

#### 2.3.2. Anthropometric Data

At enrollment, the medical history and anthropometric data were collected. All the body measurements were obtained by the same physician. Weight (in kg), height (in m), and waist circumference measurements (in cm) were used to calculate the anthropometric indices, such as the body mass index (BMI = weight in kg/height in m^2^), waist-to-hip ratio (WHR = waist circumference/hip circumference), and waist-to-height ratio (WHtR = waist circumference/height in cm), to provide additional metabolic data. The BMI cut-off of 25 kg/m^2^ was used to define BMI categories (lean vs. overweight and obese). Hirsutism was assessed with the modified Ferriman–Gallwey (FG) score in all the participants by the same physician.

#### 2.3.3. Imaging

The ovarian volume was measured by abdominal or transvaginal ultrasound with the GE Veluson S10 ultrasound system, and the cut-off of 10 cm^3^ determined the presence of PCOM [32]. Liver steatosis was assessed by an ultrasonographic evaluation of liver parenchyma echogenicity (zero, mild, moderate, or severe liver steatosis) compared to the right kidney cortex, at the Radiology Department of the Aretaieio University Hospital. A normal liver is equally echogenic to the renal cortex. When the liver echogenicity is mildly increased, it is considered grade I; when the large hepatic vessels are obscured, it is grade II; and when the diaphragmatic outline is obscured, it is grade III liver steatosis [36]. Adolescents with zero grade were characterized ‘without NAFLD’, whereas those with any other grade were classified as ‘with NAFLD’. The measurements were performed by a single designated radiologist. Measurements of the FibroScan were performed on the right lobe of the liver through the intercostal spaces on participants lying in the dorsal decubitus position with the right arm in maximal abduction. The tip of the probe transducer was covered with coupling gel and was placed on the skin between the rib bones at the level of the right lobe of the liver. The operator, assisted by an ultrasonic time–motion image, located a liver portion at least 6 cm thick that was free of large vascular structures. Once the measurement area had been located, the operator pressed the probe button to start the acquisition. The measurement depth was between 25 mm and 65 mm below the skin surface. Measurements that did not have a correct vibration shape or a correct follow-up of the vibration propagation were automatically rejected by the software. Up to 10 successful measurements were performed on each participant. The success rate was calculated as the ratio of the number of successful measurements over the total number of acquisitions. The results were expressed in kilopascal (kPa). The median value of the successful measurements was deemed to be representative of liver stiffness. The whole examination duration was less than five minutes. The liver stiffness measurements were only obtained with at least five successful measurements, and a success rate of at least 30% were considered reliable. The 95th percentile (7.9 kPa) of the study by Tokuhara et al. [37] was used as a cut-off for the diagnosis of hepatic fibrosis.

#### 2.3.4. Calculations 

The homeostatic model assessment for the insulin resistance (HOMA-IR) index was selected to assess IR and was calculated using the following formula: HOMA-IR = fasting insulin (mIU/mL) × fasting glucose (mg/dL)/405 [38]. The cut-off of 2.32, proposed by Chissini et al. [39] for pubertal females, was used to diagnose insulin resistance. The free androgen index (FAI) was calculated to determine hyperandrogenism with the following formula: FAI = 100 × [(Total Testosterone (nmol/L)/SHBG (nmol/L)] [40].

### 2.4. Bias

Every case–control study suffers from several biases. Ideally, the case and the control groups should have almost the same characteristics, such as age, gender, overall health status, and other factors. The same methodology was used to recruit cases and controls, to overcome selection bias [41]. 

### 2.5. Sample Size

The calculation of the sample size was performed before the start of the study. The design of the study was considered as pilot. The aim was to gather at least 12–25 participants per group (case and control) [42]. 

### 2.6. Statistical Analysis

A probability value of *p* ≤ 0.05 was considered statistically significant. The normal distribution was verified using the Shapiro–Wilk criterion and, graphically, with the use of histograms. Statistical analyses were performed with SPSS 26 (SPSS Inc. Chicago, IL, USA) and R version 4.1.3. The categorical data are presented as frequencies and the continuous data are presented as the mean ± standard deviation or median (interquartile range), depending on the normality test result. Comparisons were performed using the *t*-test, Mann–Whitney U test, and ANOVA with the Tukey–Kramer test for post hoc analysis. The Holm–Bonferroni method was performed to correct the *p*-values for multiple comparisons. The correlations among the quantitative data were determined with Spearman’s rho and Pearson’s r correlation coefficient. Logistic regression was used to predict the binary outcome (presence of NAFLD) based on a series of independent variables.

## 3. Results

### 3.1. Participants, Blood Parameters, Anthropometric Measurements, and Imaging

A total of 80 Caucasian adolescent females were initially enrolled in the study, 40 with PCOS and 40 controls, but five adolescent controls did not show up for the investigation. The study sample included 75 adolescent females (40 with PCOS and 35 controls) aged 12.28 to 19.08 years (mean ± SD, 15.25 ± 1.56 years). The two groups did not differ in age, but the adolescents with PCOS had a higher BMI (25.66 vs. 23.23 kg/m^2^, *p* = 0.032). The demographic, anthropometric, biochemical, hormonal, and imaging parameters of the PCOS patients and controls are listed in Table 2.

### 3.2. Main Results

Adolescents with PCOS demonstrated significantly lower SeP and SHBG concentrations than the controls (2.47 (0.40) vs. 2.66 (0.36) μg/mL, *p* = 0.025; and 41.71 ± 19.41 vs. 54.94 ± 22.12 nmol/L, *p* = 0.011). The fetuin A concentrations did not differ significantly between the PCOS patients and the controls (Table 3).

The SHBG also differed significantly between the lean and overweight/obese participants of the total sample (54.44 ± 22.68 vs. 39.25 ± 16.92 nmol/L, *p* = 0.002). No statistically significant differences in the concentrations of any of the hepatokines were found between the lean and overweight/obese controls or between the lean and overweight/obese PCOS patients. A one-way ANOVA was conducted to compare hepatokines among the four subgroups (lean controls, lean PCOS patients, overweight/obese controls, and overweight/obese PCOS patients). There was a statistically significant difference only in the SHBG (*p* = 0.005) (Table 4). Post hoc comparisons using the Tukey–Kramer test indicated that SHBG differed significantly between the lean controls and overweight/obese patients with PCOS.

Among the controls, participants with low insulin sensitivity exhibited lower SHBG concentrations than those with normal insulin sensitivity (61.18 ± 23.90 vs. 43.09 ± 17.51 nmol/L, *p* = 0.046), but this difference was not significant after correcting the *p*-values for multiple comparisons with the Holm–Bonferroni method. No statistically significant differences in the SHBG concentrations were observed among the PCOS patients. 

Higher FGF21 concentrations were observed in adolescents with PCOS and NAFLD (*n* = 7) than in those with PCOS but without NAFLD (*n* = 32) (198.80 (241.50) vs. 116.80 (84.70) pg/mL, *p* = 0.028), as shown in Table 5. No such differences were observed in SHBG, SeP, or fetuin A.

### 3.3. Correlations

In the total sample, fetuin A did not significantly correlate with age, nor with anthropometric parameters or blood pressure measurements. Similarly, no correlations were observed with liver enzymes, total cholesterol, lipoprotein concentrations, IR markers, androgen concentrations, or ovarian volume. In the total sample and in participants with low insulin sensitivity, fetuin A was significantly and negatively correlated with LDLc (r = −0.387, *p* = 0.014 and r = −0.430, *p* = 0.022, respectively). Interestingly, fetuin A was positively associated with TG (r = 0.511, *p* = 0.015) in the lean controls. A positive association between FGF21 and WHtR was found in the PCOS patients (rho = 0.372, *p* = 0.028), independently of their BMI status. In the controls, FGF21 and liver enzymes (AST, rho = −0.370, *p* = 0.048; ALT, rho = −0.444, *p* = 0.016) were negatively correlated.

We found significant correlations of SeP with testosterone and FAI (r = 0.325, *p* = 0.007 and r = 0.361, *p* = 0.002, respectively). These correlations remained in the controls and PCOS patients, and in the controls, SeP was also positively associated with ovarian volume (r = 0.414, *p* = 0.021) and WHR (r = 0.385, *p* = 0.024) but negatively associated with HDL (*p* = −0.369, *p* = 0.045).

SHBG demonstrated multiple significant negative correlations with adverse metabolic parameters both in the total sample (Table 6) and separately in the study groups. 

### 3.4. Regression Analysis

A regression analysis was performed to investigate if the hepatokines could predict the diagnosis of hepatic steatosis. It was found that a 1 pg/mL increase in the FGF21 concentration increased the odds of an NAFLD diagnosis by liver ultrasound by 1% when all the other parameters remained unchanged. The significance of the FGF21 concentrations in the NAFLD models was present in the PCOS patients and in the overweight and obese adolescents. 

Additionally, an increase of age by 1 year and an increase of HOMA-IR by 1 increased the risk for NAFLD diagnosis (*OR* = 220% and *OR* = 98%, respectively). 

## 4. Discussion

To the best of our knowledge, this case–control study is the first of its kind, since it assessed the hepatokine profile of FGF21, selenoprotein P, fetuin A, and SHBG in adolescent PCOS patients. The concentrations of SeP and testosterone were positively associated, but significantly lower concentrations of SeP were observed in PCOS adolescents when compared with their control counterparts. No differences were demonstrated in the fetuin A values between the PCOS patients and controls, but there was a positive correlation between TG and fetuin A in the lean controls. Decreased concentrations of SHBG were observed in overweight/obese (vs. lean) participants of the total sample and in overweight/obese adolescents with PCOS vs. the lean controls; however, this known relationship between SHBG and PCOS serves as a confounding factor for the relationship of SHBG with BMI and NAFLD. No significant difference was found in the FGF21 concentrations between the PCOS patients and controls; however, FGF21 was positively correlated with WHtR in PCOS patients and was associated with hepatic steatosis. 

SeP variance in metabolic diseases is vague in the literature and is not mutual between glucose and lipid metabolic diseases. Since SeP is a selenium carrier to the tissues and their concentrations are in accordance with each other until the protein saturates in high selenium levels, conclusions for selenium are extrapolated for SeP. Experimental studies have demonstrated insulin exerting an inhibitory effect on SeP secretion [43], and concurrent results have emerged from observational studies for DM [44]. Additionally, it has been demonstrated in vitro that SeP dysregulates glucose metabolism and contributes to IR in hepatocytes and myocytes via the downregulation of adenosine monophosphate-activated protein kinase (AMPK) phosphorylation [23]. In contrast, lower SeP concentrations have been reported in patients with inflammatory disorders, hepatic fibrosis, and metabolic syndrome (MetS) [44,45]. This observation can be explained by SeP glutathione peroxidase antioxidant action, its interplay with cytokines, and its role in the apoptosis of hepatic stellate cells [46]. Additionally, positive correlations between SeP and HDL were observed, along with negative correlations among SeP and BMI, WC, BP, transaminase concentrations, and HOMA IR in prepubertal children [45,47]. There are two studies that explored the association of SeP with PCOS. In 2017, Zagrodzki et al. did not find significant differences in the concentrations of SeP between PCOS patients and controls, nor a statistically significant interaction in the correlations model [48]. On the contrary, Temur et al. reported significantly higher SeP in adult PCOS patients than in the controls and an association independent of age, BMI, HOMA-IR, and total testosterone between higher SeP concentrations and PCOS [49]. 

Fetuin A is a multifunctional protein that participates in metabolism, bone mineralization, the cardiovascular system, and the central nervous system [50]. Insulin receptors are the target of fetuin A in the liver and the skeletal muscles, whereas in adipose tissue, this hepatokine suppresses adiponectin expression and promotes inflammation [51,52]. Phosphorylated fetuin A seems to inhibit the phosphorylation of the insulin receptor and its substrate [50,53]. Fetuin A seems to be linked with metabolic disorders in a vicious cycle. Abundant FFAs in obesity increase hepatic fetuin A production, and in turn, fetuin A aggravates IR [31,50]. Similarly, various studies have reported increased fetuin A concentrations in NAFLD patients [54]. Since fetuin A seems to be a key factor in IR, this hepatokine has been also studied in PCOS [55,56,57,58,59,60,61,62]. Many researchers have found increased concentrations of fetuin A in adult PCOS patients when compared with adult controls. In contrast, Diaz et al. demonstrated lower concentrations in adolescent PCOS patients than in the controls, which increased when non-contraceptive treatment was administered [59]. Increased HOMA-IR, insulin, BMI, triglycerides, total cholesterol, and LDLc concentrations were associated with increased fetuin concentrations. Moreover, positive correlations among testosterone, DHEA-S, and FAI concentrations have been reported [56,57,60,61,62]. 

FGF21 is one of the most widely studied hepatokines. FGF21 is a metabolic regulator in fasting and feeding [63]. In human and animal trials, the administration of FGF21 induces weight loss via the leptin pathway and it seems to have favorable effects on lipid and on glucose metabolism and fatty liver disease via decreased lipogenesis and inflammation [27,64]. Interestingly, high FGF21 concentrations have been associated with obesity and NAFLD, not only in adults, but also in adolescents [65,66,67]. Many researchers have studied FGF21 in adult PCOS patients because of its observed action in metabolic disturbances and proinflammatory pathways, but none in adolescents. Half of the studies about FGF21 demonstrated no differences between the patients and the controls [62,68,69,70], while others demonstrated prominent FGF21 concentrations in patients with PCOS [71,72,73,74]. This study did not demonstrate any differences in the concentrations of FGF21 between PCOS patients and healthy controls, but the FGF21 concentrations were significantly higher in patients with PCOS and NAFLD than those with PCOS without NAFLD. 

In clinical research and practice, SHBG has been widely used in PCOS cases. SHBG binds to steroid hormones and regulates their bioavailability. In turn, testosterone and insulin downregulate serum SHBG concentrations [75,76,77]. The connection between serum SHBG concentrations and liver diseases is not new, but was discovered nearly 40 years ago [78]. The association between SHBG and liver fat, together with its role in hepatic glucose production have demonstrated the significant role of SHBG in IR [79]. In 2017, a large meta-analysis reported lower SHBG concentrations in NAFLD patients than in controls, especially in women [80]. Recently, possible associations and predictive parameters for MetS components, such as DM and NAFLD, have been explored. The role of SHBG as a mediator and a biomarker for metabolic disease, independently of androgens, was recently discovered with Mendelian randomization in GWAS studies [14,81]. The pathophysiologic processes of both IR and hyperandrogenism seem to connect PCOS with NAFLD, and SHBG seems to be a key factor in both disorders [82]. Consistent with the literature, this research found multiple significant negative correlations between SHBG and adverse metabolic parameters, such as the WHtR and HOMA-IR. The results of this study contribute further knowledge to the literature about adolescents, along with other studies in children and adults [83,84].

The data are scarce in the literature regarding hepatokines and NAFLD in adolescent populations. It is common for metabolic disorders that are encountered mainly in adults to be neglected in adolescence. Moreover, adolescents are often overlooked in various studies examining both pediatric and adult populations. Regarding hepatokines in pediatric NAFLD, the findings are contradictory: higher, lower, and equal FGF21 concentrations relative to the controls have been reported [85,86,87]. A recent meta-analysis of fetuin A in NAFLD reported different results among different age categories: the increased levels of fetuin A in adult NAFLD patients compared to the controls were not replicated in children/adolescents [88]. The association of selenoprotein P and SHBG with NAFLD is more forthright and similar throughout the age ranges [83,87,89]. To clarify the role of hepatokines in metabolic disorders such as NAFLD, more studies should be conducted in different age groups.

The recent hypothesis of a hepato-ovarian axis has already been demonstrated in Mendelian randomization (MR) studies [90]. Freed from usual confounders and reverse causality bias, Liu et al. proved that women with higher genetic potential for NAFLD are more likely to develop PCOS [90]. SHBG has been proposed as a key mediator of this influential association [90]. SHBG and a plethora of other hepatokines are believed to function equally as mediators and biomarkers for metabolic diseases [14,87]. Since liver and IR seem to be the orchestrator of metabolic disorders, elucidating the connections and metabolism among hepatokines, their secretion and function in healthy and metabolically challenged individuals could result in novel diagnostic and therapeutic modalities. More studies with participants of all age groups are needed to clarify the role and value of hepatokines in everyday clinical practice. Given the established association between PCOS and the NAFLD spectrum, it would be significant to identify an initial and inexpensive biomarker for hepatic steatosis in this population.

The limitations of this study include a lack of histologic confirmation for NAFLD. A liver biopsy may be the gold standard to define liver disease; however, in children and adolescents, non-invasive testing methods are preferable, except in cases of patients who have an increased risk of advanced fibrosis [91]. Additionally, the non-invasive biomarkers and imaging are less expensive and more suitable for epidemiological studies. Regarding the study sample, there was an apparent discrepancy between the initial calculation of the sample size and the actual sample size. As this was a pilot study, the aim was to gather at least 12 to 25 participants per group (case and control) for statistical inference; however, the achieved sample was larger to ensure validity of the study and generalization of the findings. Due to the pilot nature of this study, the sample size was not adequate for receiver operating characteristic (ROC) curve estimation, as the predictive model used in this study was representative for this sample but not for the general population [92].

## 5. Conclusions

SeP and SHBG serum concentrations were found to be significantly decreased in PCOS adolescent patients, and SeP was significantly and positively correlated with androgens. Additional to SHBG’s known actions in PCOS, this hepatokine demonstrated multiple significant negative correlations with adverse metabolic parameters. Among the PCOS patients, the FGF21 concentrations were significantly higher in those with NAFLD, whereas a 1 pg/mL increase in the FGF21 concentration increased the odds of NAFLD by 1%, suggesting FGF21 as a potential biomarker for hepatic disease in females with PCOS in adolescence. Fetuin A was the least differentiated hepatokine between the PCOS patients and controls, with the least associations with metabolic and hormonal parameters. 

## Figures and Tables

**Table 1:** The Rotterdam criteria and the recommendations of the Pediatric Endocrine Societies for the diagnosis of PCOS in adolescence.

Rotterdam Criteria (2 out of 3)	2015 Pediatric Endocrine Societies	2017 Pediatric Endocrine Societies
Clinical and/or biochemical hyperandrogenism (HA)	Moderate to severe hirsutism Persistent, poor response to topical acne therapy Persistent elevated levels of serum total and/or free testosterone	Evidence of HA (required) Biochemical Clinical (progressive hirsutism)
Oligo- or anovulation	Menstrual intervals persistently <21 days or >45 days, 2 or more years after menarche Consecutive menstrual intervals >90 days, regardless of years after menarche Lack of menses by 15 years or 2–3 years after thelarche	Persistent irregular menses/oligomenorrhea (required)
Polycystic ovarian morphology (PCOM)	Deferring diagnostic evaluation for PCOM ovarian volume > 12 cm^3^ could be considered	Optional criteria Severe cystic acne PCOM
Exclusion of other etiologies of androgen excess and anovulation		Two years post-menarche; rule out other disorders of hyperandrogenism

HA: hyperandrogenism; PCOM: polycystic ovarian morphology.

**Table 2:** Demographic, anthropometric, biochemical, hormonal, and imaging data in adolescents with PCOS and controls, and in subgroups of PCOS patients defined by BMI cut-off of 25 kg/m^2^.

				PCOS		
	PCOS (*n* = 40)	Controls (*n* = 35)	*p*-Value	Lean (*n* = 19)	Overweight/Obese (*n* = 21)	*p*-Value
Age (years)	15.45 ± 1.59	15.00 ± 1.49	0.211	15.36 ± 1.56	15.54 ± 1.65	0.733
Menarche (years)	11.87 ± 1.36	11.60 ± 0.82	0.312	12.56 ± 1.13	11.21 ± 1.25	**0.001**
BMI (kg/m^2^)	25.66 ± 5.30	23.23 ± 4.19	**0.032**	21.27 ± 2.25	29.64 ± 3.92	**<0.001**
BMI percentile by age ^§^	89.50 (32.80)	78.50 (39.70)	0.130	63.00 (30.30)	94.00 (4.85)	**<0.001**
FG Score ^§^	12.00 (5)	4.00 (9)	**<0.001**	12.00 (5)	12.00 (7)	0.791
SBP (mmHg)	116.63 ± 10.53	112.09 ± 7.86	**0.050**	112.05 ± 8.94	120.73 ± 10.34	**0.011**
DBP (mmHg)	67.69 ± 9.80	65.84 ± 7.41	0.394	65.18 ± 11.05	70.05 ± 8.09	0.157
WC (cm)	76.71 ± 9.90	72.63 ± 8.04	0.057	69.13 ± 4.40	83.21 ± 8.59	**<0.001**
WHtR	0.47 ± 0.06	0.45 ± 0.05	0.185	0.42 ± 0.035	0.51 ± 0.05	**<0.001**
WHR	0.74 ± 0.06	0.74 ± 0.71	0.883	0.72 ± 0.05	0.76 ± 0.05	**0.010**
ALT ^§^ (U/L)	14.00 (11)	13.00 (6)	**0.017**	13.00 (8)	20.00 (12)	0.060
AST ^§^ (U/L)	17.50 (5.25)	16 (5)	0.164	17.00 (5)	18.00 (6)	0.750
gGT ^§^ (U/L)	11.90 (8)	10 (5.65)	**0.037**	10 (4)	16 (9)	**0.010**
TC ^§^ (mg/dL)	149.10 (32.52)	146.50(40.85)	0.675	149 (37.80)	149 (28.50)	0.708
HDLc (mg/dL)	53.96 ± 13.15	58.10 ± 11.23	0.165	56.91 ± 12.24	51.29 ± 13.66	0.180
LDLc ^§^ (mg/dL)	84.00 (34.40)	83.40 (33.70)	0.885	77.10 (34.40)	89.80 (36.70)	0.573
ApoA1 (mg/dL)	135.81 ± 19.51	142.80 ± 33.75	0.564	143.00 ± 22.55	128.62 ± 13.76	0.073
ApoB ^§^ (mg/dL)	76.80 (19.25)	58.00 (43.50)	0.538	74.60 (22.50)	79 (23.50)	0.999
LpA ^§^ (nmol/L)	14.50 (52.50)	12.10 (18.88)	0.389	15.15(127.55)	13.50 (44.70)	0.497
TG ^§^ (mg/dL)	66.65 (49.68)	60.65 (29)	0.964	51.00 (36.60)	72.50 (46.50)	**0.027**
Glucose (mg/dL)	87.38 ± 7.75	83.35 ± 6.04	**0.020**	87.74 ± 9.21	87.05 ± 6.36	0.783
Insulin ^§^ (μU/mL)	11.15 (7.69)	9.55 (7.32)	0.098	8.95 (4.31)	14.25 (9.67)	**0.006**
FSH (mIU/mL)	5.15 ± 1.91	5.41 ± 2.25	0.597	4.86 ± 1.75	5.40 ± 2.05	0.384
LH ^§^ (mIU/mL)	5.91 (7.68)	2.95 (2.45)	**0.003**	5.91 (8.48)	6.3 (7.7)	0.478
E2 ^§^ (pg/mL)	39.15 (24.50)	28.00 (21.39)	**0.028**	39 (28.50	42 (26)	0.663
PRL^§^ (ng/mL)	12.53 (8.23)	12.85 (8.72)	0.567	12.7 (10.35)	12.30 (6.99)	0.839
Testosterone ^§^ (ng/mL)	0.41 (0.27)	0.32 (0.20)	**0.002**	0.41 (0.35)	0.42 (0.24)	0.728
FAI ^§^	3.51 (4.06)	2.19 (1.91)	**0.001**	2.76 (2.81)	4.45 (5.64)	**0.028**
DHEA-S (μg/dL)	251.57 ± 109.68	246.42 ± 98.34	0.837	253.91 ± 83.15	249.57 ± 130.27	0.904
D4 ^§^ (ng/dL)	2.89 (1.53)	2.77 (1.85)	0.170	3.24 (1.74)	2.56 (1.71)	0.064
25OH-VitD (ng/mL)	26.48 ± 9.22	27.05 ± 7.62	0.805	25.14 ± 8.69	27.83 ± 9.92	0.487
Ovarian volume (cm^3^)	11.59 ± 3.82	5.52 ± 2.22	**<0.001**	11.19 ± 4.58	11.94 ± 3.11	0.547
FibroScan stiffness (kPA)	6.25 ± 1.54	6.87 ± 3.61	0.570	5.60 ± 1.45	6.66 ± 1.45	0.245
HOMA-IR ^§^	2.28 (1.55)	1.86 (1.37)	**0.025**	1.95 (1.00)	2.89 (2.61)	**0.015**

PCOS: polycystic ovary syndrome; BMI: body mass index; FG: Ferriman–Gallwey; SBP: systolic blood pressure; DBP: diastolic blood pressure; TC: total cholesterol; TG: triglycerides; FSH: follicle-stimulating hormone; LH: luteinizing hormone; E2: estradiol; PRL: prolactin; FAI: free androgen index; DHEA-S: dehydroepiandrosterone sulfate; D4: androstenedione; 25OH-VitD: 25-hydroxyvitamin D; HOMA-IR: homeostatic model assessment-insulin resistance. Values refer to mean ± standard deviation and *t*-test or ^§^ median (interquartile range) and Mann–Whitney U. Bold numbers indicate statistically significant differences.

**Table 3:** Hepatokine descriptive statistics of the entire study sample and in PCOS patients and controls.

	All Adolescents (*n* = 75)			PCOS (*n* = 40)	Controls (*n* = 35)	
	Min	Max	Mean ± SD or Median (IQR)	Mean ± SD or Median (IQR)	Mean ± SD or Median (IQR)	*p*-Value
Fetuin A (g/L)	0.46	1.34	0.94 ±0.15	0.94 ± 0.16	0.94 ± 0.16	0.972
FGF21 ^§^ (pg/mL)	4.40	460.00	117.30 (106.65)	90.00 (110.40)	127.20 (87.80)	0.077
SeP ^§^ (μg/mL)	1.95	3.88	2.60 (0.39)	2.47 (0.40)	2.66 (0.36)	**0.025**
SHBG (nmol/L)	4.32	110.00	47.74 ± 21.58	41.71 ± 19.41	54.94 ± 22.12	**0.011**

PCOS: polycystic ovary syndrome; Min: minimum; Max: maximum; SHBG: sex hormone-binding globulin; FGF21: fibroblast growth factor 21. Values refer to mean ± standard deviation and *t*-test or ^§^ median (interquartile range) and Mann–Whitney U. Bold numbers indicate statistically significant differences.

**Table 4:** Concentrations of hepatokines by BMI categories in PCOS and control groups.

	PCOS (*n* = 40)			Controls (*n* = 35)			*p*-Value (Comparison among 4 Categories)
	Lean (*n* = 19)	Overweight/ Obese (*n* = 21)	*p*	Lean (*n* = 24)	Overweight/ Obese (*n* = 11)	*p*	
Fetuin A (g/L)	0.96 ± 0.15	0.92 ± 0.17	0.523	0.95 ± 0.16)	0.92 ± 0.12	0.659	0.892 ^#^
FGF21 ^§^ (pg/mL)	77.30(113.85)	108.20 (103.70)	0.404	136.77 ± 67.25	150.27± 77.60	0.613	0.405 *
SeP ^§^ (μg/mL)	2.44 (0.36)	2.59 (0.48)	0.320	2.73 (0.37)	2.61 (0.17)	0.885	0.098 *
SHBG (nmol/L)	47.53 ± 20.41	36.77 ±17.52	0.093	60.04 (44.22)	44.22 (15.26)	0.061	**0.005** ^#^

PCOS: polycystic ovary syndrome; BMI: body mass index; SHBG: sex hormone-binding globulin; FGF21: fibroblast growth factor 21; SeP: selenoprotein P. Values refer to mean ± standard deviation and *t*-test or ^§^ median (interquartile range) and Mann–Whitney U. * Kruskal–Wallis was used for non-parametric and ^#^ ANOVA for parametric comparisons. Bold numbers indicate statistically significant differences.

**Table 5:** Concentrations of hepatokines by NAFLD diagnosis in PCOS group.

	PCOS with NAFLD (*n* = 7)	PCOS without NAFLD (*n* = 32)	*p*
Fetuin A (g/L)	0.99 ± 0.10	0.93 ±0.17	0.404
FGF21 ^§^ (pg/mL)	198.80 (241.50)	116.80 (84.70)	**0.028**
SeP ^§^ (μg/mL)	2.70 (0.54)	2.45 (0.35)	0.761
SHBG (nmol/L)	35.70 ± 16.80	43.12 ±19.96	0.370

PCOS: polycystic ovary syndrome; NAFLD: non-alcoholic fatty liver disease; SHBG: sex hormone-binding globulin; FGF21: fibroblast growth factor 21; SeP: selenoprotein P. Values refer to mean ± standard deviation and *t*-test or ^§^ median (interquartile range) and Mann–Whitney U. Bold numbers indicate statistically significant differences.

**Table 6:** Correlations of hepatokines with anthropometric, metabolic, and hormonal parameters in the total sample.

	*n*	r	*p*-Value
**SHBG and**			
Age	68	−0.321	0.008
	35 (controls)	−0.337	0.037
BMI	68	−0.398	<0.001
	35 (controls)	−0.492	0.005
SBP	62	−0.299	0.018
	28 (controls)	−0.429	0.023
WHtR	67	−0.407	<0.001
	31 (controls)	−0.373	0.039
	36 (PCOS)	−0.406	0.014
TC	67	−0.276	0.024
	37 (PCOS)	−0.341	0.039
Testosterone	66	−0.295	0.016
Ovarian volume	66	−0.312	0.011
	40 (PCOS)	−0.349	0.037
HOMA-IR	62	−0.400	0.001
	34 (PCOS)	−0.455	0.015
**SeP and**			
Testosterone	68	0.325	0.007
FAI	68	0.361	0.002

## Data Availability

The research data are available upon request.
